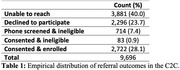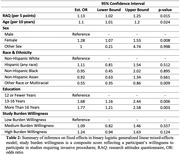# Effectiveness of a Local Recruitment Registry

**DOI:** 10.1002/alz.086986

**Published:** 2025-01-09

**Authors:** Adam I Birnbaum, Zion T Grant‐Freeman, Joshua D Grill, Dan Hoang, Adrijana Gombosev, Daniel L Gillen

**Affiliations:** ^1^ University of California, Irvine, Irvine, CA USA; ^2^ Institute for Memory Impairments and Neurological Disorders, University of California, Irvine, Irvine, CA USA

## Abstract

**Background:**

Recruitment registries are tools to decrease the time and cost required to identify and enroll eligible participants into clinical research. Despite their potential to increase the efficiency of accrual, few analyses have assessed registry effectiveness. We investigated the outcomes of study referrals from the Consent‐to‐Contact (C2C) registry, a recruitment registry at the University of California, Irvine.

**Method:**

We categorized the outcome of study referrals as: (1) unable to reach, (2) declined to participate, (3) phone screened but ineligible, (4) consented but ineligible or (5) consented and enrolled. We assessed overall effectiveness of the registry via the empirical distribution of referral outcomes. To assess associations between participant characteristics and referral outcomes we used a binary logistic generalized linear mixed‐effects model with study‐specific random intercepts, restricting the analysis to the outcome of the first study referral for each participant in the subset of referrals for which we had complete subject‐level data (n = 3,238). We treated any referral leading to obtaining participant consent as a success. We tested for an association between the odds of successful first referral and score on the research attitudes questionnaire (RAQ) via a 2‐sided Wald test.

**Result:**

The C2C made 9,696 referrals to 60 studies. 2,805 (28.9%) of these referrals were successful, leading to participant consent. In 40% of referrals, the participant did not respond to contact and in 23.7% of referrals the participant declined. We estimated that participants who scored 5 points higher on the RAQ had 13% higher odds of successful first referral compared to otherwise similar participants (95% CI: 1.02,1.25, p = 0.015). Other factors significantly associated with successful enrollment included older age (OR = 1.10 per 10 years, 95% CI: 1.01,1.2, p = 0.024), female sex (OR = 1.28, 95% CI: 1.07,1.53, p = 0.008), and higher education (ref≤12 years; 13‐16 years: OR = 1.68, 95% CI: 1.16,2.44, p = 0.006; >16 years: OR = 1.77, 95% CI: 1.21,2.58, p = 0.003).

**Conclusion:**

These results suggest that the C2C is an effective tool in aiding accrual in ADRD clinical research and that the RAQ may be a meaningful predictor of referral outcomes.